# Supplementary material for: Polyketide synthase gene clusters in the ericoid mycorrhizal fungus Oidiodendron maius: genome-wide prediction, expression profiling, and functional characterization of knockout mutants
Source: Front Plant Sci. 2026 Sep 16;17:1924430. doi: 10.3389/fpls.2026.1924430 (PMC13623903; doi:10.3389/fpls.2026.1924430)
Supplement: Supplementary file 1 [file SupplementaryFile1.zip › Supplementary Materials/Supplementary Folder 1.zip!/Oidma1_AssemblyScaffolds_Repeatmasked/knownclusterblast/cluster29/jgi.p_Oidma1_44028_mibig_hits.html]

| MIBiG Protein | Description | MIBiG Cluster | MiBiG Product | % ID | % Coverage | BLAST Score | E-value |
| --- | --- | --- | --- | --- | --- | --- | --- |
| AIG62133.1 | 6-methylsalicylic\_decarboxylase | BGC0000120 | Patulin\_biosynthetic\_gene\_cluster | 47 | 93.3526011561 | 292 | 4.9e-79 |
| EHA22194.1 | amidohydrolase\_2 | BGC0000170 | Yanuthone\_D\_biosynthetic\_gene\_cluster | 44 | 97.1098265896 | 264 | 1.9e-70 |
